# First-Time Mothers’ Expectations and Experiences of Postnatal Care in England

**DOI:** 10.1177/1049732320944141

**Published:** 2020-09-17

**Authors:** Jenny McLeish, Merryl Harvey, Maggie Redshaw, Jane Henderson, Reem Malouf, Fiona Alderdice

**Affiliations:** 1 University of Oxford, Oxford, United Kingdom; 2 Birmingham City University, Birmingham, United Kingdom

**Keywords:** postpartum care, reproduction, users’ experiences, health care, mothers, mothering, families, maternity, nursing, qualitative, longitudinal trajectory analysis, United Kingdom

## Abstract

Postnatal care is the aspect of maternity care with which women in England are least satisfied. Little is known about first-time mothers’ expectations of postnatal care, or how these expectations relate to their experiences and appraisal of care. Thirty-two first-time mothers took part in a longitudinal qualitative descriptive study, based on two semi-structured interviews—the first in pregnancy, and the second 2 to 3 months after birth. Trajectory analysis was used to identify the thematic patterns in the relationships between postnatal care expectations, needs, experiences, and confidence. Five trajectories were identified, showing that mothers’ satisfaction with postnatal care and confidence were primarily influenced not by the extent to which their expectations were met but the varied extent to which their individual postnatal needs were met. Rapid and responsive assessment of needs both antenatally and postnatally, and appropriate adjustment of care, is key in supporting women effectively at this time.

## Background

Postnatal care has an important role in enabling a safe and successful transition to parenthood (Demott et al., 2006), but is the aspect of maternity care with which women in England are least satisfied (Redshaw & Henderson, 2015). In surveys and qualitative studies, some women have reported negative experiences of the physical environment of postnatal wards, staff attitudes and communication, staff having insufficient time to offer meaningful support, lack of information about baby care, and inadequate support for infant feeding (Malouf et al., 2019). The consequences of poor postnatal care may be especially problematic for first-time mothers, who need to develop parental confidence and skills (Barclay et al., 1997; Leahy-Warren & McCarthy, 2011), and this may be particularly challenging for mothers who are young, socioeconomically disadvantaged, or lack social support at home (Kurtz Landy et al., 2008). Timely support from professionals offered as part of postnatal care can help new mothers to develop this confidence (Leahy-Warren, 2005; Wiegers, 2006), which is inversely associated with stress, anxiety, and postnatal depression (Leahy-Warren & McCarthy, 2011).

Little is known about pregnant women’s expectations of postnatal care. It has been suggested that some of the dissatisfaction women express in relation to their postnatal care may be attributable to a mismatch between their expectations and their experiences, and that this may be particularly acute for women from Black, Asian, and other ethnic minority communities (Beake et al., 2010; Jomeen & Redshaw, 2013; Puthussery et al., 2010). The role played by expectations in patient satisfaction has been much debated, with some models proposing that expectations are an important component in satisfaction (Jackson et al., 2001; Linder-Pelz, 1982), while others argue that the significance of met or unmet expectations is eclipsed by factors such as appraisal of actual experiences (Pascoe, 1983; Thompson & Suñol, 1995).

Expectations are not a single concept, but can be divided into four different constructs (Thompson & Suñol, 1995): ideal (wants, hopes, preferences), predicted (real, anticipated outcomes), normative (what ought to happen), and unformed (where the person does not have any expectations). Researchers investigating expectations and experiences of postnatal care have not always distinguished clearly between these different forms of expectations, nor between the expectations of first-time mothers and those with previous experience of postnatal care, who may have different needs (Forster et al., 2008; Lindberg et al., 2008). Pregnant women’s *ideal* expectations for postnatal care have been reported in a small number of studies, along with limited information about their *real* expectations (Forster et al., 2008; Hirst & Hewison, 2002; Lindberg et al., 2008). In other studies, women have been asked retrospectively about their *real* expectations of postnatal care (Beake et al., 2010; Bowling et al., 2012; Forster et al., 2008; Puthussery et al., 2010). This is not necessarily a reliable way of exploring expectations, because after an event, people may adjust or misremember their expectations of it (Scott & Alwin, 1998; Thompson & Suñol, 1995), and Miller (2000) has shown how first-time mothers retrospectively reconstruct their narratives of becoming a mother. Alternatively, *ideal* or *normative* expectations have been inferred by researchers from women’s answers to questions about postnatal experiences (Jomeen & Redshaw, 2013; Ockleford et al., 2004; Puthussery et al., 2010; Redshaw & Henderson, 2012).

This article explores what first-time mothers *really* expected during pregnancy from their postnatal care, what they actually experienced, the influence of their prior expectations on their appraisal of postnatal care, and the extent to which it affected their parental confidence. It reports research that is part of a program of work, including an online survey and cross-sectional analyses of mothers’ real and ideal expectations and experiences, which will be reported separately.

## Method

### Study Design

This was a longitudinal qualitative descriptive study (Sandelowski, 2000; Thomson & Holland, 2003), based on semi-structured, in-depth interviews, and underpinned by a contextualist approach which emphasizes sequence and process within experiences (Madill et al., 2000; Mjøset, 2009). Qualitative longitudinal research focuses on process and change over time (Hermanowicz, 2013; Thomson & Holland, 2003). Data may be analyzed by repeat cross sections—pooling cases to compare themes at different time points, or longitudinally—following individual trajectories over time (Grossoehme & Lipstein, 2016; Thomson & Holland, 2003). This article uses longitudinal trajectory analysis with a qualitative descriptive design, as the purpose of the study was to explore the relationship between participants’ own expectations and subsequent experiences, and thus to stay close to their accounts without imposing a theoretical framework or generating theory (Tindall, 1994). At the same time, this study acknowledges the roles of both participants’ understandings and the researchers’ interpretations in the production of knowledge (Madill et al., 2000). Throughout the research process, the researchers worked with a reflexive awareness of their own perspectives on the transition to motherhood and postnatal care, based on professional knowledge and diverse personal experiences. The University of Oxford Medical Sciences Inter-divisional Research Ethics Committee approved the study.

### Recruitment

The criteria for participation were that women were currently in the third trimester of pregnancy; aged 16 or over; planning to give birth in England; and had not given birth previously. Purposive maximum variation sampling (Patton, 2002) was used to recruit women with a range of sociodemographic characteristics, with a particular emphasis on seeking diversity in age, socioeconomic status, and ethnicity. Three recruitment routes were used: (a) an online survey about expectations of postnatal care, promoted on social media by parent support organizations, at the end of which information was provided about the qualitative longitudinal research with an invitation for women to leave contact details if they wished to be considered; (b) an in-person invitation from a researcher to women attending three sessions of a young mother’s antenatal group and two sessions of a free antenatal exercise class, each run by a community group in a different area of high deprivation; and (c) an advertisement circulated on social media by a multiple birth charity. The researchers had no prior contact with any of the participants.

### Data Collection

Data were collected through two semi-structured qualitative interviews: the first when the participant was in the last 3 months of pregnancy, and the second 2 to 3 months after birth. First interviews were carried out by McLeish and Harvey between October 2017 and March 2018, and second interviews between January and July 2018. Topic guides for the interviews are given in Additional Files 1 and 2 of Supplementary Material. Women for whom the research team had contact details were telephoned or emailed to confirm their willingness to participate in the research. If they agreed, they were emailed a participant information sheet and consent form at least 24 hours before the interview, which was carried out by telephone. Women with whom the researcher had face-to-face contact were given a participant information sheet and consent form and offered the options to take part in a face-to-face interview at a time and place of their choice, or a telephone interview.

Informed consent was obtained at the beginning of the interview through a signed consent form if face-to-face, or given verbally and recorded in writing if by telephone. Participants consented to data collection and for their interview responses to be used in publications with no details being published that could identify them. At the end of the first interview, participants were asked for permission for the same researcher to contact them approximately 6 weeks after their baby’s due date to arrange a second interview. Participants were offered a shopping voucher worth £10 at the end of the first interview and a shopping voucher worth £15 at the end of the second interview, to thank them for their time. All interviews were carried out in English, although interpreting support was available if required. Interviews were audio-recorded and fully professionally transcribed. No other adults were present at face-to-face interviews.

In longitudinal qualitative research, it is usual to overrecruit at the initial stage, to allow for the likelihood that some participants will be lost to follow-up (Hermanowicz, 2013; Thomson & Holland, 2003). Data collection in this study therefore continued past the point where data saturation was reached in first interviews, to allow for subsequent drop out and ensure demographic variation.

### Data Analysis

Longitudinal trajectory analysis (Grossoehme & Lipstein, 2016) was carried out for the participants who completed both interviews, and focused on between-case analysis, where each case was the repeated interviews of a single participant over time (Calman et al., 2013). Longitudinal trajectory analysis is usually carried out and presented for a small number of individual participants, because of the high volume of data generated through repeat interviews (Calman et al., 2013; Thomson, 2007). This analysis sought to identify the thematic patterns in the relationships between expectations, experiences, and confidence of *all* participants.

Transcripts were checked against the audio-recording, and read and reread for familiarity. Data from all participants were charted into a matrix on an Excel spreadsheet, condensed into summarized form with key verbatim quotations incorporated (Ritchie & Lewis, 2003). The Y-axis was organized by participant. The X-axis was organized by broad topics derived from the research question: “Hospital/birth center expectations,” “Hospital/birth center experiences,” “Community expectations,” “Community experiences,” “Postnatal support needs,” and “Impact on confidence.” Within these topics, summaries were created through inductive analysis of participants’ experiences, including both anticipated and unanticipated issues. To assist between-case analysis, additional columns were used on the X-axis to classify (a) the participant’s expectations of postnatal care as high, low, or mixed, and specific or unformed; (b) her self-described postnatal support needs as high or low; (c) her experiences of care as high, low, or mixed; and (d) her self-described confidence at the time of second interview as high or low. Where there was a consistent pattern identified between expectations, experiences, and outcomes for a number of participants, these were grouped together as a single trajectory. Particular attention was paid to negative cases that were anomalous to the emerging patterns. Two researchers (McLeish and Alderdice) carried out the trajectory analysis independently, discussing and agreeing the classifications used and the trajectories.

When the expectations, experiences, and outcomes of all participants had been grouped into trajectories, the details from the matrix were used to identify individuals who appeared to most clearly exemplify each trajectory. Where there was variation within a trajectory, more than one participant was identified to represent this variation. Participants were given pseudonyms and personally identifying details were removed.

## Results

### The Participants

Forty pregnant women took part in first interviews, at gestations between 27 and 40 weeks (median: 38 weeks). Thirty-two first interviews were by telephone and eight were face-to-face, ranging in length from 12 to 75 minutes (mean: 31 minutes). Thirty-two women (80%) took part in second interviews, when their babies were 7 to 15 weeks old (median: 11 weeks); eight women could not be contacted after birth. Twenty-nine second interviews were by telephone and three were face-to-face, ranging in length from 21 to 56 minutes (mean: 37.5 minutes).

The results reported here are based on the 32 women who took part in both interviews. Background information about them is shown in Supplementary Table 1. The eight mothers who were not contactable for second interviews were predominantly younger (six were below 25 years old), more ethnically diverse (three identified as Black or Asian), and living in more disadvantaged areas (all were in the two most deprived quintiles), using the Index of Multiple Deprivation (Ministry of Housing, Communities and Local Government, 2015). There were no differences in the results identified between participants interviewed by telephone and those interviewed face-to-face.

### Expectations, Experiences, and Confidence: Trajectories

Across the 32 participants, expectations of postnatal care did not appear to shape their appraisal of care as actually experienced. For many women, these antenatal expectations were so unformed that there was little relationship with subsequent experiences of postnatal care. Others had developed more specific expectations, with some expecting high levels of postnatal support and some expecting low levels of support. Some women had high expectations because they knew in advance that they would have additional needs, for example, as a young mother, as a mother of twins, or as a mother living with physical or mental illness. Others only identified a need for a higher level of support postnatally when they encountered unforeseen difficulties, for example, with breastfeeding, with their baby’s health, or with their own physical or mental health. Whatever the mother’s expectations of care, the strongest influence on her satisfaction with postnatal care and her self-described parental confidence was not whether these expectations were met but rather the extent to which her actual postnatal needs for support were met.

**Figure 1.**
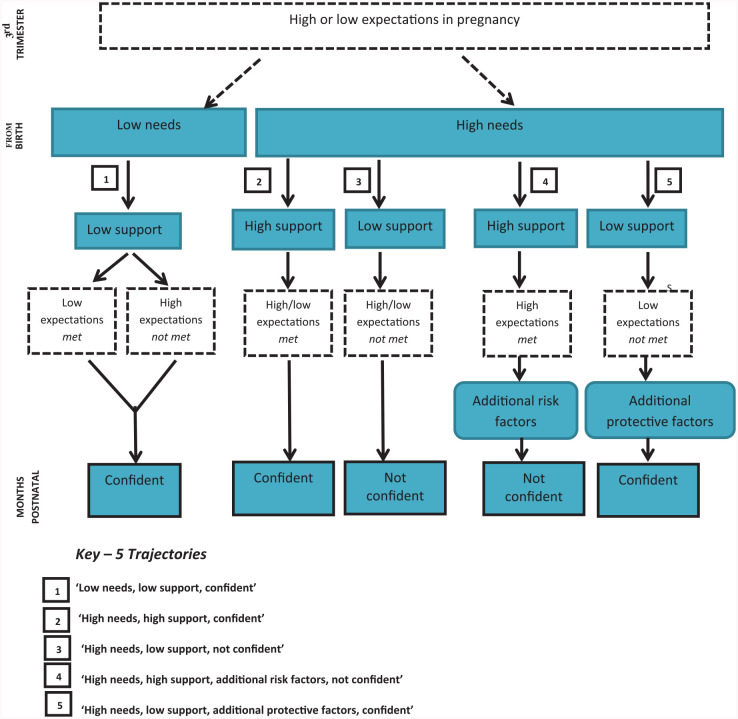
Trajectories between expectations, needs, experiences, and postnatal confidence.

Five trajectories, shown in Figure 1, were identified for all 32 women in the interplay between expectations, needs, experiences, and the mother’s confidence. There were three predominant trajectories (Trajectories 1–3) and two that were less common (Trajectories 4 and 5). As the level of a woman’s expectations had such a limited impact on her experiences, these trajectories are organized around the differences in the level of support needed, support received, and the impact on the woman’s self-described parental confidence. The differences between high and low expectations are explored within the descriptions of each trajectory.

The anonymized cases of eight mothers have been used to illustrate the five identified trajectories. The first three common trajectories are each represented by two mothers to illustrate the variation based on differing levels of antenatal expectations. The last two, less common, trajectories are each represented by one mother.

#### Trajectory 1: “Low Needs, Low Support, Confident” (Amy—low expectations and Beth—high expectations)

In this trajectory, women had high or low expectations of postnatal care, and low postnatal support needs. They had received basic support proportionate to their needs and had become confident mothers. Women who had low expectations of care, and received a low level of care, were satisfied because it met both their needs and expectations. Women who had high expectations of care were disappointed in the low level of care they received. However, because it met their needs, they did not feel they had received inadequate care, nor that it had negatively impacted their transition to motherhood.

Amy, who had a planned home birth, was an example of a mother who had limited expectations of postnatal care and felt that the support was better than she expected. In her antenatal interview, she thought that there might be a single home visit from a midwife or health visitor on the day after birth. She was pleased to have three home visits from midwives, and then a visit from a health visitor which was much more positive than her expectations: “It was far more about helping us out, rather than checking up on us . . . I thought it [would be] a bit more social services type thing, like seeing if there was anything that we were doing wrong.” She attended weighing clinics once a month where she was able to ask her questions. Amy said she felt confident right from birth, and she summarized her experience of postnatal care very positively in the light of how well routine care had met her straightforward needs: “Definitely got the right amount of support available, it’s up to you whether you want to use it.”

Beth had high and specific expectations of “lovely” hospital postnatal care: “Help with feeding. Obviously I’ll have the C-section, help with lifting the baby. And any mental health support.” Following a cesarean section, she at first received the attentive support she had expected on the postnatal ward: “If I pressed the bell someone was there within a couple of minutes to help me.” In other respects during her 3-day stay, her experience was less good than her high expectations. The ward environment was not restful with babies crying and upset mothers: “I didn’t get any sleep . . . It wasn’t ideal.” Beth felt confused by conflicting information from staff about feeding and swaddling: “It was all a bit contradictory, when you’ve not done it before. I didn’t know who’s right.” She found it stressful that her husband was not allowed to stay overnight and wished she had been warned to expect this: “It was difficult without him . . . I could have prepared myself mentally if I’d have known.”

In the community, Beth had expected home visits, and she was disappointed that after one visit from a midwife, subsequent appointments were at a clinic, which she found difficult to manage shortly after her cesarean section: “We weren’t too pleased . . . Luckily my husband was still off, otherwise I don’t know what I’d have done.” It was also harder than she expected to get postnatal appointments with her General Practitioner (GP): “It felt a bit like I’d just been left, once I’d been discharged from the midwife.” Nonetheless, Beth had recovered easily from the cesarean, had no difficulty breastfeeding, and described herself as confidently enjoying motherhood. While Beth’s high expectations of postnatal care had not been met, she felt the care had been good enough: “We can’t complain really . . . I suppose I’ve not really had any issues, and if I had more issues it might have been more of a problem.”

#### Trajectory 2: “High Needs, High Support, Confident” (Evie—low expectations; Cristina—high expectations)

In this trajectory, women had high or low expectations of care, and had high postnatal support needs because of physical or mental health challenges, the baby’s health problems, or difficulties breastfeeding. They had received high levels of support that both met their needs and met or exceeded their expectations. This led to high satisfaction with care and had enabled them to develop parental confidence despite the challenges they faced.

Evie had negative expectations of “scary” hospital postnatal care, based on her friends’ reports of “bossy” midwives. She found staff attitudes much better than she had expected: “I never really felt pushed aside or belittled.” The staff were responsive whenever she asked for help, although she did wonder whether this might be because she and her baby were clinical priorities: “I had quite a lot of medical needs, and [my baby] was being quite closely monitored, so they had a lot more time for me.”

Evie had mostly unformed and low expectations about postnatal care in the community: she was anticipating disempowering interactions with health professionals, because she had heard that midwives could be patronizing, health visitors judgemental, and GPs cynical. Her actual experience was quite different. She was pleasantly surprised to find that the “very friendly” midwives did home visits, and regretted not knowing this in advance, “because maybe that would have made me feel a bit less worried.” Her health visitor, who made herself available by telephone and text message as well as in person, turned out to be “very kind, not very intrusive . . . She gives all the things she has to give you but in a very non-lecturey way.” Evie had expected that professional reassurance would be important to her after birth, but she thought it was unlikely that she would get it. In fact, she found the support available exactly met her needs in this respect: “I think it’s made me very confident because I’ve been able to ask all those silly questions when I needed to, so you’re not constantly wondering, ‘Am I doing this right?’”

By contrast with Evie, Cristina had well-defined high expectations about practical and emotional support in hospital. When her twins were born, she received a high level of support from staff, but breastfeeding did not go well, which at the time felt to her like failure. She praised the way a busy midwife responded to her emotional distress: “She really took time, actually sitting down on the end of my bed, giving all those cues of, ‘I’m here and I’m listening and I’m not trying to rush out of the door’.” Overall she felt that her 5 days in hospital had been very useful: “I was by then feeling quite confident . . . the support that I had while I was there was really key.”

Cristina had expected that as the mother of twins she might get additional support from professionals in the community. These expectations were fulfilled, with extra home visits and easy access to the health visitor: “They would always come to us, I was never expected to have worked out how to get out and about with [the babies] . . . I can ring my health visitor at any time and ask her questions.” Although it had been challenging for Cristina to come to terms with eventually giving up breastfeeding, her confidence had been boosted by affirmation from her health visitor: “The reassurance that my babies are doing okay and that I was managing okay and I was doing a good job.” Overall Cristina felt that her postnatal care had both exceeded her high expectations and had met her high needs: “It’s been better than I was expecting. When I was pregnant I don’t think I really realised the care that I might need afterwards. All that follow-up care that’s been given has been really excellent.”

#### Trajectory 3: “High Needs, Low Support, Not Confident” (Gabi—low expectations; Faith—high expectations)

In this trajectory, women had high or low expectations of care and high support needs. They had received inadequate support that did not meet their needs and were not confident. Women who had high expectations of care were disappointed in the care they received, because it had not met their needs or expectations. Women whose low expectations of care were met were nonetheless dissatisfied with the care received because it had not met their needs. Both Gabi (who had low expectations) and Faith (who had high expectations) had unresolved difficulties with breastfeeding; and they had lost confidence in themselves as mothers.

Gabi had only vague expectations of hospital care (“I don’t really know anything about it. I’m not really sure what my expectations are”) and her experience was “a bit of a nightmare” until (in line with her expectations) she was able to transfer to a freestanding birth center for extra help with breastfeeding. Before moving there she had two unpleasant, sleepless nights on a postnatal ward, which was hot and noisy, while the busy midwives did not have time to see if their suggestions about breastfeeding were effective. Her husband was sent home when she arrived on the ward, and she was left on her own overnight, feeling abandoned and disorientated:

*I was expecting somebody to come back and show me where the toilet was and just orientation stuff . . . I didn’t know whether I had to take the baby with me or leave him in his cot, or how I was meant to go to the toilet. Because I had this tiny life clinging to my chest and I just didn’t know what to do.*

Gabi also had low, largely unformed expectations about postnatal care in the community. The confidence she had built up in the birth center was undermined by a lack of support for her ongoing difficulties with feeding. She found the health visitors’ clinics inhospitable to new mothers because they were held first thing in the morning, in a noisy playgroup which did not allow pushchairs and had nowhere to sit: “If baby is sleeping you have to walk around with your baby in your arms until they can see you.” It was also difficult to contact the health visitor when Gabi needed her help urgently: “There were a few times when we were really at our worst and struggling where I phoned up and couldn’t get through . . . and it would be at least 24 hours before they were able to return a call.” Contradictory advice from professionals had led her to doubt her own judgment:

*One piece of advice I was given was, “Don’t do what people tell you to do, trust your instincts,” but I felt like my instincts were non-existent by the time I was given that advice because we’d been given so many different conflicting bits of information*.

Frequent repeated weighing of her baby to monitor growth, without effective support to resolve feeding problems, had made her feel judged: “I still feel under immense pressure . . . I feel like all the time I’m being scrutinised as my ability as a mother.” Gabi had received no wider reassurance to affirm her competence in the face of these difficulties, and her parenting confidence had collapsed: “I basically doubt every single thing I do with him.” She wished she could have built a relationship of trust with a key professional: “It needs to be the same person that sees you each time so that you have consistency of care . . . you become more comfortable with them and they are more aware of your situation.”

Faith’s stay in hospital was much worse than she expected. Her friends had suggested that that it was good to spend some time on the labor ward after birth because there was plenty of help offered, but in reality she felt abandoned for 3 hours after birth with no information: “We were like, ‘What are we meant to do?’ Because we didn’t have a baby, now we’ve got a baby.” She was expecting nurturing care on the postnatal ward to enable both parents to “have a bit of a bubble around them while they’re in this nice, shiny, new baby mode . . . creating that bond as a family.” She spent three nights there, and although she had anticipated it would be noisy and busy, she had not imagined the exhaustion and stress this would cause her and her partner: “Very confused . . . chaotic . . . a really loud, stressful, noisy, difficult environment.” She was expecting and needed advice on looking after her baby and breastfeeding, but found the way that it was delivered in hospital to be contradictory and disempowering. The reality was that the midwives did not have time for her and she did not feel able to be assertive in asking for help:

*They would say, “Oh yes, I’ve just got to go do one thing and I’ll be back immediately.” They’re so busy that they go back into the corridors and get called away by 10 other people, so it would be about three or four hours sometimes, or more, before you’d see them again.*

Faith had no clear expectations about community postnatal care. She found that community midwives were unreliable and hard to contact, and lost trust in their advice after they directly contradicted each other. She liked her health visitor, but was left with many questions about baby care which she did not feel able to ask: “It’s not really been how to be a parent.” Her difficulties with breastfeeding continued and anxiety about her baby’s slow weight gain, coupled with pressure from professionals to introduce formula milk, was intensely stressful. She believed antenatally that she had realistic expectations about becoming a mother: “I certainly don’t expect to be a perfect mum straight away, and probably not ever.” However, her experience of confusing, unreliable, and insufficient postnatal care had left her “struggling,” and she reflected that “different or better feeding support would have just really changed our lives.”

#### Trajectory 4: “High Needs, High Support, Additional Risk Factors, Not Confident” (Holly—high expectations)

In this trajectory, women had high expectations of care, had high support needs, received high levels of support, and were satisfied with their care which met their expectations. However, due to social or mental health complexity, this high level of support had not enabled them to become confident mothers.

Holly, who was a young, single mother, had not formed any expectations at all about postnatal care in hospital: she was focused on birth and when she thought about the time after birth it was in practical, personal terms: “I don’t have to walk around with extra weight . . . you get to bring your own clothing.” Holly had to stay in hospital for a week after birth, and found this time “very boring,” although the staff were “friendly.” Her mother stayed with her, looking after her and the baby and supporting her to breastfeed.

Holly’s postnatal experiences in the community were dominated by the fact that when discharged from hospital she was required to live at a mother and baby foster placement; if she did not pass a parenting assessment, her baby would be removed from her care. She described the experience of being a new mother while living away from her own mother and being constantly assessed, as “sad . . . strange . . . stressful.” She was expecting home visits from her teenage pregnancy midwife and a health visitor, although she was not sure how long the midwifery care would continue: “[The midwife] said she’ll be with me forever, so . . . but I think that was a joke.” These expectations were borne out in practice. She did not rate information from the midwife or health visitor as particularly useful, because “most of my family members, they have babies already, so I already know quite a lot.” She was, however, careful to seek out and follow the advice of health professionals, to increase her chances of passing the assessment:

*I confirm, as in I always go to my mum and then I’ll just double check [with the professionals], so say for example [the baby] reacts badly to whatever I’ve done, I can be like, “Well, we all confirmed it.”*

Like other young mothers in this study, she found it difficult to balance appropriate advice-seeking with demonstrating parenting competence. The foster carer’s role was experienced as an uncomfortable blend of assistance and judgment: “It’s got to be done their way, not your way . . . [they] set you up to fail, because they expect you to know how to do it already.” Despite the high level of support she received, Holly found the realities of motherhood overwhelming: “You know when after you’ve given birth you just want the baby? When he was sleeping, I just wanted to wake him up. But then, a month after, you just want him to sleep . . .”

#### Trajectory 5: “High Needs, Low Support, Additional Protective Factors, Confident” (Jacqui—low expectations)

In this trajectory, women had low expectations of care, and had high support needs. They did not receive the support they needed, and were dissatisfied with their care. Unlike the women in Trajectory 3, they had nonetheless become confident parents through the support of family or their own existing self-efficacy, which buffered the impact of inadequate professional support.

Jacqui did not expect much from her hospital care: “I think they would probably check if I’m okay with feeding and then just send me off.” Likewise, she had limited expectations of care in the community, which she knew would involve midwives and a health visitor but not when, where, or why; she said that if she encountered a problem, she expected to ask for their help or “look it up, or just sort of handle it.” The care she received during a brief hospital stay of less than 24 hours did not even meet these low expectations:

*I only had people coming in doing the exit stuff with me . . . I did ask a couple of midwives to help me settle [my baby] . . . and they just suggested a couple of things and then went away and didn’t really help.*

When discharged, Jacqui was far from “okay with feeding”: “I had quite a lot of nerve pain in my breast and my back, I had blood coming from my nipples and they were shredded to pieces.” She appreciated that a midwife and the health visitor came out to check on her, but described their care as “quite minimal”; they had their own agendas and did not give her useful support, and she was referred to a breastfeeding group at a time when she could barely leave the house. Jacqui had been “on the verge of giving up” breastfeeding but, unlike Faith and Gaby, inadequate professional support did not destroy her confidence in her own judgment. She did her own research and found her own solutions, noting that this went against the advice she was given: “The NHS hospitals aren’t very keen on how I solve my own problems . . . but I think the best support I got was just finding out for myself.” She had gone on to establish breastfeeding successfully, but she felt that this was despite, not because of, her postnatal care which had “made it a bit more of a rocky start.”

## Discussion

This study illustrates how first-time mothers in England had varied expectations of postnatal care during the third trimester of pregnancy, ranging from low to high and from unformed to specific. They also had a wide range of subsequent experiences of care, ranging from positive to negative, some of which met or exceeded their expectations and some of which disappointed them. In repeat cross-sectional studies of expectations and experiences, it is impossible to go beyond these generalizations (Hirst & Hewison, 2002), but trajectory analysis has enabled the links between expectations, experiences, appraisal of care, and parental confidence to be tracked for individuals and then analyzed for common patterns, reported as five trajectories.

This trajectory analysis has demonstrated that, as argued by Pascoe (1983) and Thompson and Suñol (1995), and as reported by Hirst and Hewison (2002), predicted expectations did not significantly shape satisfaction with postnatal care for any of the mothers, even though most did not give birth in their preferred setting. Instead this was primarily influenced by the extent to which the care received met the mother’s actual postnatal needs, which were not necessarily foreseeable in pregnancy. Mothers could be both disappointed that postnatal care did not meet high expectations and yet satisfied with it as meeting need. Conversely having low expectations of care did not make it easier to cope when these expectations were realized, for example, expectation that the postnatal ward would be noisy and busy did not help a mother to deal with the resulting exhaustion and lack of staff attention. This is in line with Pascoe’s conceptualization of health care satisfaction as a person’s cognitive evaluation of, and affective response to, the context, process, and result of the care they receive (Pascoe, 1983). Pascoe argues that cognitive evaluation involves comparison against an individually identified standard, in this case the mother’s subjective perception of the extent to which care received was proportionate to her needs. In line with the findings reported in a recent review (Walker et al., 2019), the key aspects of mothers’ evaluations were timely availability of access to appropriate support when needed and the quality of the interactions with the health professionals providing care. In particular, mothers experienced these interactions as positive when the support received from a professional was closely attuned to their individual needs at that time, a process described by Smythe et al. (2014) as “tactful practice.” Some mothers said that ideally they would like continuity of care during the postnatal period, to build a relationship. Only five of the mothers received at least one postnatal contact from a midwife who they had also met during pregnancy, but for most this was a pleasant surprise rather than an expectation.

This is not to argue that it is unimportant for women to have accurate information during pregnancy about postnatal care. Mothers who had limited advance information about postnatal care worried unnecessarily that they would be unsupported. Some mothers from varied sociodemographic backgrounds expressed negative expectations about judgmental health visitors, which sometimes contrasted with subsequent positive experiences. Canvin et al. (2007) have highlighted how these negative expectations may have significant consequences for some disadvantaged mothers, whose fears about being judged or reported to social services lead them to not engage with the health visiting service.

This study provides evidence in support of the contention that high-quality, needs-led professional support in the postnatal period plays a key role in enabling first-time mothers to make a confident transition to parenthood by providing positive feedback and direct support to master skills such as breastfeeding (Forster et al., 2008; Leahy-Warren, 2005; Walker et al., 2019). Trajectory 2 shows how high support matched to high need can enable mothers to overcome the challenges they face, while Trajectory 3 shows that a lack of support, or high *quantity* of support contacts experienced by mothers as low *quality*, can undermine their ability to develop their parental confidence. Unresolved challenges with breastfeeding were prominent as an obstacle to developing confidence and maternal emotional well-being in this trajectory. This is consistent with evidence that successful breastfeeding is associated with better maternal mental health for women who plan to breastfeed (Borra et al., 2015), and that women whose maternal identity is closely connected with breastfeeding may need sensitive support to cope with unsuccessful breastfeeding experiences (Sheehan et al., 2013).

These findings also illustrate the importance of high-quality postnatal care across the care continuum between hospital or birth center and the community, in the context of the reduced length of average time spent by new mothers on a postnatal ward (Bowers & Cheyne, 2016; Nilsson et al., 2015). The contrasting community experiences of mothers in Trajectories 2 and 3 suggest that in some areas, the health visiting “Universal Plus” offer of increased support to mothers with higher needs (Public Health England, 2018) was being successfully delivered, but in other areas this was failing. Although there has been targeted government funding in England to develop perinatal mental health services (NHS England, 2018), this has not been matched by investment in ordinary postnatal care, which has the potential to prevent some mental health problems from developing (Borra et al., 2015; Leahy-Warren & McCarthy, 2011).

Trajectories 4 and 5 illustrate exceptions to the general pattern. Trajectory 5 demonstrates how for some women, the impact of insufficient postnatal care could be buffered by social support in the community, or mitigated by strong pre-existing personal self-efficacy. This is consistent with Young and Roberts’ findings in their review of the application of resilience theories in the transition to parenthood (Young et al., 2019), and it emphasizes the importance of seeing postnatal care in the wider context of mothers’ efforts to seek out and navigate postnatal information and support from other mothers and online (Price et al., 2018). Trajectory 4 shows the opposite situation: there were some women whose needs were so complex that they were not met even by a level of postnatal care which the mothers themselves considered good. This suggests the importance of postnatal care being embedded in a joined-up, multi-agency response to vulnerable mothers’ needs, and indicates the benefits of the sustained, intensive support given to young mothers in programs such as the Family Nurse Partnership, which may improve maternal self-efficacy (Robling et al., 2016).

Trajectory 4 also shows the limitations of postnatal care in supporting the development of a mother’s skills and confidence where there are safeguarding concerns about her ability to care for the baby. Mother and baby foster carers generally lack specialist training or guidance and may be unclear about the expected parameters of their role (Knight et al., 2006; November & Sandall, 2020). A study of teenage mothers in foster care demonstrated the variability in foster carers’ willingness and ability to provide effective emotional support for young mothers during the transition to parenthood (Mantovani & Thomas, 2015). Given the challenges that most first-time mothers face in developing their own parenting confidence when they are *not* separated from their partners and support networks, it may be questioned whether the most effective way to enable vulnerable women to become competent and confident parents is through placement with a carer without specialist training. Further research could investigate ways in which postnatal care can be most effectively adapted to meet the individual needs of mothers and babies who are vulnerable or have additional needs, and how postnatal care professionals can work with professionals and organizations outside the health service to improve the care offered to new mothers in the community.

One strength of this research was the participation of 32 women from across England and from a variety of sociodemographic backgrounds. The qualitative longitudinal design using trajectory analysis enabled the rich, in-depth exploration of the relationship between women’s expectations about postnatal care and subsequent experiences, appraisal of care, and outcomes. It was a limitation that the stronger diversity of the original sample of 40 was diluted because mothers who were lost to follow-up were younger, more socioeconomically disadvantaged, and more ethnically diverse than those who took part in second interviews. In addition, three of the first interviews were unusually short (15 minutes or less), limiting their depth. These participants were all young pregnant women (aged below 18 years) who had not formed any expectations of postnatal care and found it challenging to engage with questions about what might happen in the future. All three were lost to follow-up, so are not included in the analysis presented here.

## Conclusion

Longitudinal qualitative research using trajectory analysis is a useful and feasible method of exploring real predicted expectations and subsequent experiences in the perinatal period. First-time mothers’ satisfaction with postnatal care and their confidence as new mothers were primarily influenced not by the extent to which their expectations were met but the varied extent to which their postnatal needs were met. Rapid and responsive assessment of needs both antenatally and postnatally, and appropriate adjustment of care, is key in supporting women effectively at this time.

## Supplemental Material

Additional_file_1 – Supplemental material for First-Time Mothers’ Expectations and Experiences of Postnatal Care in England Click here for additional data file. Supplemental material, Additional_file_1 for First-Time Mothers’ Expectations and Experiences of Postnatal Care in England by Jenny McLeish, Merryl Harvey, Maggie Redshaw, Jane Henderson, Reem Malouf and Fiona Alderdice in Qualitative Health Research

Additional_file_2 – Supplemental material for First-Time Mothers’ Expectations and Experiences of Postnatal Care in England Click here for additional data file. Supplemental material, Additional_file_2 for First-Time Mothers’ Expectations and Experiences of Postnatal Care in England by Jenny McLeish, Merryl Harvey, Maggie Redshaw, Jane Henderson, Reem Malouf and Fiona Alderdice in Qualitative Health Research
