# Supplementary material for: First-Time Mothers’ Expectations and Experiences of Postnatal Care in England
Source: Qual Health Res. 2020 Sep 17;30(12):1876–87. doi: 10.1177/1049732320944141 (PMC7528544; doi:10.1177/1049732320944141)
Supplement: Additional_file_1 – Supplemental material for First-Time Mothers’ Expectations and Experiences of Postnatal Care in England [file Additional_file_1.pdf]

Topic Guide for women's expectations of postnatal care (1st interview)

1. **Background**
   - Age, ethnicity, gestational age, employment, education, location (if not known from survey)
   - Can you tell me a bit about the antenatal care you're getting from midwives or doctors now? (e.g. care from team/one-to-one, health issues, multiple birth)

2. **Thinking about postnatal care**
   - Could you tell me a bit about where your ideas on postnatal care in hospital or birth centre have come from?
     - How helpful have you found that?
     - How well informed do you feel?
   - How much do you want to know about postnatal care when you're pregnant?
     - How easy do you find it to take in information about what it will be like after birth?
     - When would you ideally like information about postnatal care?
     - And other information about postnatal life?

3. **Expectations of postnatal care in hospital or birth centres**
   - **What do you think it will be like staying on the ward after birth?**
     - Roughly how long do you expect to stay?
     - What do you think the ward will be like?
     - How staff will treat you after you've had a baby?
   - **What sort of help are you expecting to get from staff?**
     - Different if caesarean?
   - **What are you expecting will be allowed for visitors and partners?**
     - What do you expect the rules on visitors will be?
     - Do you expect/want your partner to be able to stay overnight?
     - How involved do you expect your partner to be?
   - **Who do you expect to decide when it's time to go home?**

- **Do you have any other expectations you would like to add?**

4. **Expectations of postnatal care at home**
   - **What professional care are you expecting once you're home?**
     - Who, where, how often?
   - **What sort of help are you expecting?**
   - **Who else do you expect will be helping you?**
     - And in what ways?
   - **Do you have any other expectations you would like to add?**

5. **In an ideal world…**
   - If it was up to you, what sort of postnatal care do you think women *should have* in hospital/birth centre and at home?
     - who
     - where
     - when
     - what

6. **When you think about life after your baby is born, how are you feeling about becoming a parent?**
