# Supplementary material for: First-Time Mothers’ Expectations and Experiences of Postnatal Care in England
Source: Qual Health Res. 2020 Sep 17;30(12):1876–87. doi: 10.1177/1049732320944141 (PMC7528544; doi:10.1177/1049732320944141)
Supplement: Additional_file_2 – Supplemental material for First-Time Mothers’ Expectations and Experiences of Postnatal Care in England [file Additional_file_2.pdf]

Topic Guide for women's experiences of postnatal care (2nd interview)

1. **Could you tell me about your birth and a little bit about what life has been like for you since?**
   - Getting used to life with a baby
   - Your physical health / recovery
   - Your confidence / coping
   - Your emotions
   - How have things been for your partner?

2. **What was the postnatal care like in hospital/ birth centre?**

   - **What was it like staying on the ward after birth?**
     - How long did you stay, and was that the right amount of time for you?
     - Who decided when it was time to go home?
     - What was the ward like?
     - How did staff treat you?

   - **What sort of help did you get from staff & was this what you needed/wanted?**
     - Did you have to ask for help/information, or was it offered?
     - Did you feel as involved as you wanted to be in any decisions about your care or your baby's care?
     - Was there any information/help you would have liked but didn't get, or questions you didn't feel able to ask?
     - Was all the information consistent?

   - **What were the rules for visitors and partners, and how did these work for you?**
     - Did your partner feel included as much as they wanted?

   - **Overall, what sort of impact would you say the postnatal care in hospital had on you?**

3. **What was the postnatal care like at home?**
    - **What professional care did you have once you were home?**
        - E.g. who, where, how often? Any midwife who looked after you in pregnancy?
        - E.g. Were you given the contact details of a midwife you could call when you were discharged, and if so did you call and was that helpful?
        - How did these professionals treat you?
    - **What sort of help did you get and was this what you needed/ wanted?**
        - Was there any information/help you would have liked but didn't get, or questions you didn't feel able to ask?
        - Was all the information consistent?
    - **What other support or help did you get (eg from family/friends)**
        - What was that like?
        - How did that help?
    - **Overall, what sort of impact would you say the postnatal care at home had on you?**
4. **Thinking back to when you were pregnant, how does what really happened in your postnatal care compare to what you were expecting or imagining?**
    - **[USE FIRST INTERVIEW]**
    - **Is there anything know now that you wish you had known then?**
5. **Best and worst**
    - What was the best thing about your postnatal care?
    - What was the worst thing?
    - To what extent do you feel that the care was adapted to your own individual circumstances or needs?
6. **In an ideal world…**
    - **What sort of postnatal care do you think women *should have* in hospital/birth centre and at home? (What are the ingredients of good care?)**
